# Superior loco-regional control after primary surgery compared to chemo-radiotherapy for advanced stage laryngeal cancer

**DOI:** 10.3389/fonc.2023.1132486

**Published:** 2023-08-01

**Authors:** Mohamed Shelan, Lukas Anschuetz, Adrian Schubert, Beat Bojaxhiu, Daniel M. Aebersold, Olgun Elicin, Roland Giger

**Affiliations:** ^1^ Department of Radiation Oncology, Inselspital, Bern University Hospital, University of Bern, Bern, Switzerland; ^2^ Department of Otorhinolaryngology, Head and Neck Surgery, Inselspital, Bern University Hospital, University of Bern, Bern, Switzerland

**Keywords:** advanced laryngeal cancer, surgery, radiotherapy, loco-regional control, overall survival

## Abstract

**Objective:**

The optimal strategy to treat loco-regionally advanced squamous cell carcinoma of the larynx (LSCC) remains to be defined. The goal of this single institution retrospective study was to report on oncologic outcome of advanced LSCC treated with curative intent.

**Methods:**

Patients diagnosed and treated for stage T3-T4a LSCC between 2001 and 2014 were retrospectively analyzed. Time-to-event endpoints were calculated beginning from the date of histologic diagnosis, which were analyzed with log-rank test and Cox proportional hazard models.

**Results:**

The cohort was divided into two subgroups: primary radiotherapy with concomitant cisplatin (CRT) (n=30, 38%) and primary surgery (n=48, 62%). Median follow-up was 56 months. Locoregional control (LRC) for the primary surgery and CRT were 95% and 50% in 5 years, respectively (p<0.01). Progression free survival (PFS) for the primary surgery and CRT were 61% and 38% in 5 years, respectively (p=0.23). The overall survival (OS) after primary surgery and CRT in 5 years were 63% vs. 65%, respectively (p=0.93). The 5-years LRC was significantly superior after surgery compared to RT for cT3 primaries (100% vs 50%, p= 0.0022). No significant differences were observed in the remaining subgroups regarding cT stage and PFS or OS.

**Conclusion:**

Our series demonstrated superior LRC after primary surgery followed by risk-adapted adjuvant (C)RT compared to primary CRT in cT3 LSCC, but no significant difference in PFS or OS in locally-advanced LSCC. The optimal patient selection criteria for the ideal treatment for loco-regionally advanced LSCC still needs to be defined.

## Introduction

Squamous cell carcinoma is the most predominant histolopathological type of malignant laryngeal tumor (1, 2), where tobacco and alcohol overconsumption are the main risk factors. The increased awareness of those factors resulted in a 2.4% annual decrease in the incidence of laryngeal cancer in the last decade (3). However, about 40% of the cases are still diagnosed in advanced stages III and IV (Union for International Cancer Control (UICC) (4), resulting in a poor 5-year overall survival (OS) below 50% (3, 5) with a slight improvement over the last 10 year (6). It is preferred to choose an optimal treatment strategy delivering the maximal disease-free survival, while preserving the larynx and its function if possible.

In the last decades, both primary radiotherapy (RT) and primary surgery for early UICC stage I-II squamous cell carcinoma of the larynx (LSCC) were well established as acceptable modalities providing excellent oncological outcome and quality of life (7–11). On the other hand, advanced UICC stage III-IV LSCC with or without regional lymph node metastasis resembles a heterogeneous group with a complex management, where the optimal treatment strategy remains unclear (6, 12, 13) For example, the NCCN guidelines recommends upfront surgery for T4a, and there is more controversy for T3 tumors population. Still, there is a lack of modern phase III trials comparing different treatment modalities for advanced LSCC.

The aim of this single institution retrospective chart review is to report the oncologic outcome of stage III-IVB patients (T3-T4a N1-N3b) treated with curatively intended primary non-surgical and surgical treatment modalities and compare it to published literature.

## Materials and methods

After obtaining the approval from the ethics committee (Cantonal Ethics Committee of Bern – reference number: 117/14), a retrospective chart review on all patients with T3-T4a, N0-N3b, M0 primary LSCC treated in curative intent either with primary surgery or cisplatin-based combined chemo-RT (CRT) at our tertiary referral head and neck anticancer center between 2001 and 2014 was conducted. Staging was revised and adapted according to the 7^th^ edition of the UICC staging system. Exclusion criteria were histology other than squamous cell carcinoma, usage of non-cisplatin based CRT regimen, presence of distant metastatic disease or second primary malignancies at the time of or prior to the diagnosis of LSCC.

All time-to-event intervals were calculated based on the date of initial biopsy confirming LSCC. The follow-up time was not censored at any time point. The time-to-event outcomes were calculated based on the date of diagnosis, and depicted and evaluated by Kaplan-Meier curves and log-rank test, respectively. In order to isolate the adverse factors influencing the outcome parameters, multivariate Cox proportional hazard models with variables yielding p values <0.1 via univariate analyses were built, and backwards elimination was performed. Statistical analysis was done with JMP software (version 14.0 SAS Institute, Cary, NC, USA). Statistical significance was set to a two-tailed alpha of <0.05. The actuarial rates and risk estimations are provided with 95% confidence intervals (CI).

## Results

Totally, 78 patients fulfilled the inclusion criteria for the analysis. Median follow-up was 50 months (Range: 5-212). Details of patients’ demographic and disease characteristics are provided in **Table 1**. Based on primary treatment modality, the cohort was divided into two groups: primary CRT (n=30, 38%) and surgery to the primary tumor (n=48, 62%).

**Table 1:** Patients’ demographic, disease and treatment characteristics.

Parameter	Total Number of Patients (relative frequency)		
	Entire Cohort (n = 78)	Primary CRT (n = 30)	Primary Surgery (n=48)
Sex^#^			
Male Female	66 (78%)	26 (87%)	40 (83%)
	12 (22%)	4 (13%)	8 (17%)
Age (years) (median: 63)#			
≤63 >63	40 (51%)	18 (60%)	22 (46%)
	38 (49%)	12 (40%)	26 (54%)
Tumor localization*			
Supraglottic	32 (41%)	17 (57%)	15 (31%)
Glottic	10 (13%)	6 (20%)	4 (8%)
Subglottic	5 (6%)	0 (0%)	5 (10%)
Transglottic	31 (40%)	7 (23%)	24 (50%)
cT stage*			
3	42 (54%)	21 (70%)	21 (43%)
4a	36 (46%)	9 (30%)	27 (56%)
cN stage^#^			
0	37 (47%)	11 (37%)	26 (55%)
1	10 (13%)	6 (20%)	4 (8%)
2a	2 (3%)	0 (0%)	2 (4%)
2b	12 (15%)	2 (6.5%)	10(21%)
2c	14 (18%)	9 (30%)	5 (10%)
3	3 (4%)	2 (6.5%)	1 (2%)
UICC (7^th^ ed.) stage^#^			
III	26 (33%)	12 (40%)	14 (30%)
IVA	49 (63%)	16 (53%)	33 (68%)
IVB	3 (4%)	2 (7%)	1 (2%)

*: p value <0.05 in Chi-square test comparing the subgroups treated with primary surgery and RT.

**^#^:** p value >0.05 in Chi-square test comparing the subgroups treated with primary surgery and RT.

CRT, concomitant chemo-radiotherapy; ND, neck dissection; RT, radiotherapy; UICC, Union for International Cancer Control.

Out of 30 patients underwent the CRT group, only 6 (20%) patients underwent upfront neck dissection followed by CRT. Intensity modulated delivery technique (IMRT) was used in 72% of cases. The median delivered dose was 72 Gy (range: 64-76 Gy). All IMRT plans were static-field IMRT until November 2007 and then VMAT plans were used.

The majority of the primary operated patients (80%) underwent total laryngectomy, while 5% underwent partial open laryngectomy and in 5% transoral laser surgery was performed. In total, 38 patients in the surgery group received adjuvant RT with a median dose of 66 Gy (range: 60-74 Gy).

**Figure 1:**
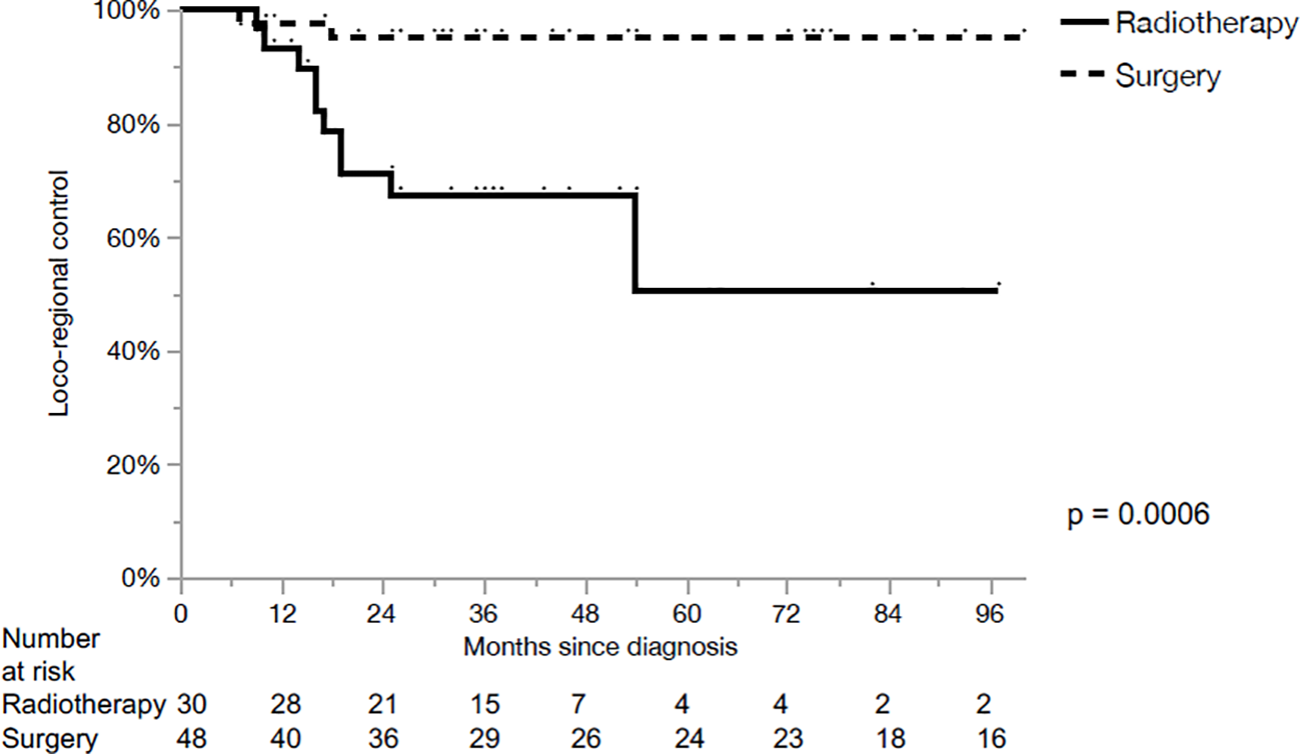
Loco-regional control.

**Figure 2:**
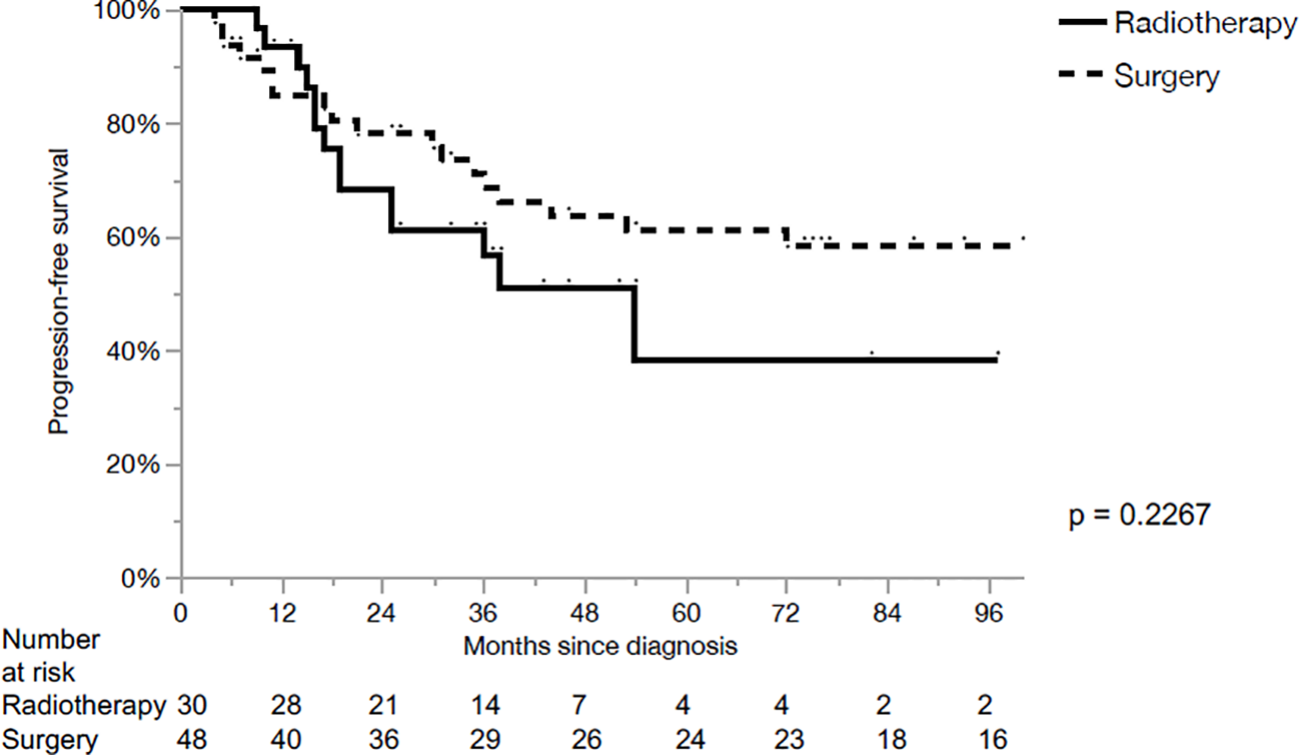
Progression-free survival.

**Figure 3:**
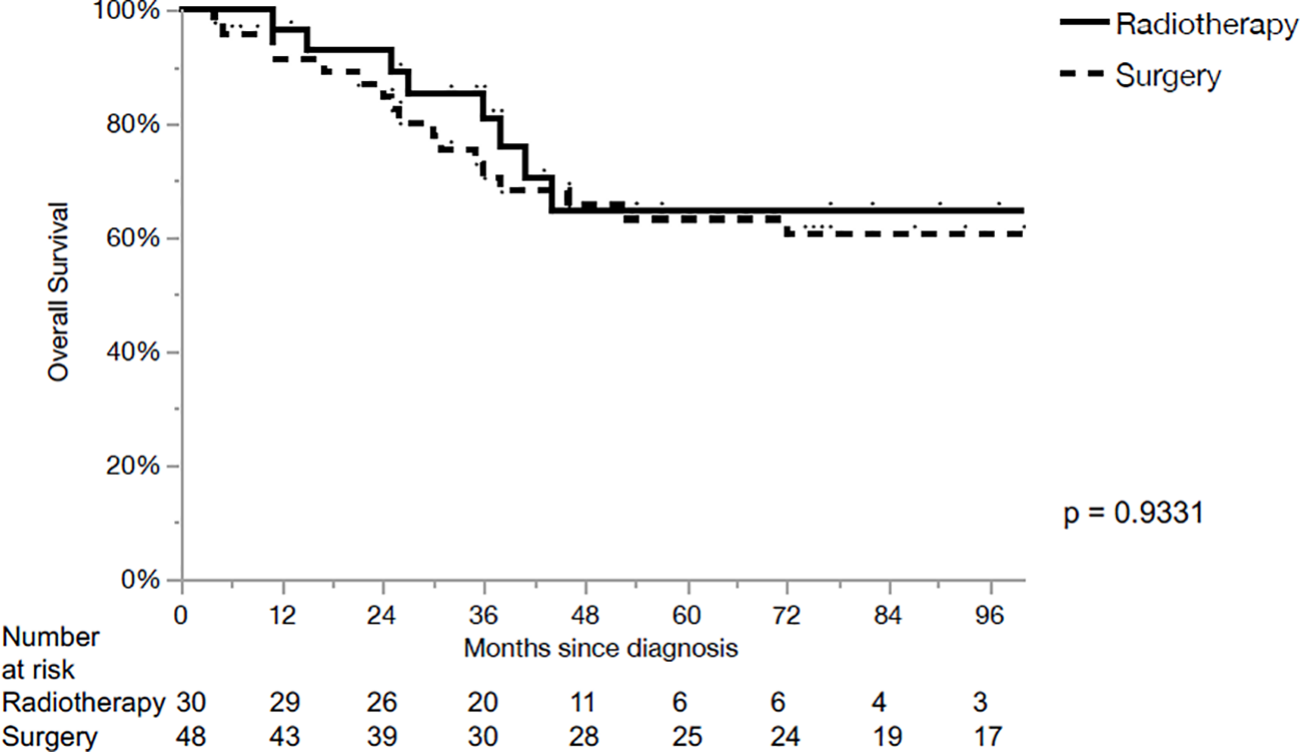
Overall survival.

The 5-year loco-regional control (LRC), progression-free survival (PFS) and OS for the whole cohort were 80%, 54% and 64%, respectively. LRC for the primary surgery and CRT groups were 95% and 50% in 5 years, respectively (p<0.01, **Figure 1**). PFS for the primary surgery and CRT groups were 61% and 38% in 5 years, respectively (p=0.23, **Figure 2**). The OS after primary surgery and CRT in 5 years were 63% vs. 65%, respectively (p=0.93) (**Figure 3**).

The 5-year outcomes based on the primary treatment modality are summarized in **Table 2**. LRC was significantly superior in the surgical group for cT3 primaries when compared with CRT (100% vs 50%, p=0.0022). However, no significant differences in PFS or OS were observed, when comparing both subgroups based on the treatment modality.No parameters influencing PFS (**Table 3**) or OS (**Table 4**) were identified.

**Table 2:** Five-year oncologic outcome with primary surgery and primary chemoradiotherapy in cT3 and cT4 cases regardless of cN.

	5-year LRC		5-year PFS		5-year OS	
Primary treatment	cT3	cT4	cT3	cT4	cT3	cT4
Surgery +/- adjuvant CRT	100%	91%	70%	54%	70%	58%
CRT	50%	67%	39%	50%	71%	47%
p value	0.0022	0.1135	0.0578	0.9768	0.6900	0.8108

CRT, concomitant chemo-radiotherapy; LRC, loco-regional control; OS, overall survival; PFS, progression-free survival.

**Table 3:** Cox proportional hazards models for progression-free survival.

Variable	Univariate	
	HR (95% CI)	p
Female vs. male	0.46 (0.16-1.34)	0.1543
Age >63 vs. ≤63	1.32 (0.68-2.57)	0.4160
cT4 vs. cT3	1.33 (0.68-2.61)	0.4060
cN≥2 vs. cN0-1	1.06 (0.54-2.08)	0.8619
Surgery +/- adjuvant CRT vs. CRT	0.65 (0.32-1.32)	0.2327
Localization		
Glottic	reference	
Supraglottic	1.41 (0.39-5.08)	0.5968
Subglottic	1.57 (0.26-9.63)	0.6270
Transglottic	2.55 (0.73-8.95)	0.1439

CI, confidence interval; CRT, concomitant chemo-radiotherapy; HR, hazard ratio.

**Table 4:** Cox proportional hazards models for overall survival.

Variable	Univariate Analyses	
	HR (95% CI)	p
**Female vs. male**	0.35 (0.10-1.20)	0.0957
**Age >63 vs. ≤63**	1.49 (0.70-3.17)	0.3017
**cT4 vs. cT3**	1.11 (0.53-2.33)	0.7884
**cN≥2 vs. cN0-1**	1.04 (0.46-2.35)	0.9333
Surgery vs. CRT		
Localization		
**Glottic**	1.13 (0.24-5.37)	0.8694
**Supraglottic**	2.31 (0.32-16.57)	0.4060
**Subglottic**	3.03 (0.70-13.16)	0.1386
**Transglottic**	0.35 (0.10-1.20)	0.0957

CI, confidence interval; CRT, concomitant chemo-radiotherapy; HR, hazard ratio.

Patterns of recurrence after the primary and subsequent salvage therapies and their treatments are shown in **Table 5**. The median follow-up after curatively-intended salvage treatment (surgery +/- RT) was 62 months (range: 52-71). Both the 5-year PFS and OS after salvage treatment were 60%.

**Table 5:** Patterns of tumor recurrence and their treatments.

Parameter	First recurrence N (crude %)	Second recurrence after salvage therapies N (crude %)
**Tumor recurrences**	17 (22%)	2 (12%)
local	9 (53%)	0
loco-regional	4 (24%)	0
distant metastasis	4 (24%)	2 (100%)
Treatment of recurrences		
Salvage surgery	3 (18%)	0
Salvage surgery + RT	2 (12%)	0
Palliative chemotherapy	8 (47%)	0
Palliative RT	1 (6%)	1 (50%)
Best supportive care	3 (18%)	1 (50%)

RT, radiotherapy.

During treatment and follow-up, a total of 65 Patients (83%) needed a percutaneous endoscopic gastrostomy (PEG) tube. At last follow-up, 47 (60%) patients underwent total laryngectomy and/or had tracheotomy.

Total laryngectomy-free survival (LFS) in 5 years was 31%. LFS after primary CRT and primary surgery were 50% and 19%, respectively (p<0.01).

Among the whole cohort, 14 patients (18%) were diagnosed with metachronous second primary malignancies (SPM) with no significant difference based on the primary treatment modalities (p=0.3934) or based on whether RT was any part of the treatment (p=0.4556). Of those, 3, 10 and 1 patients had head and neck, non-head and neck and both (synchronous) SPM, respectively. The actuarial incidence rates of SPM within 2, 5 and 10 years were 7%, 15% and 20%, respectively.

## Discussion

The main finding of our series was the higher LRC (95% vs. 50% at 5 years) with no significant differences in PFS or OS after primary surgery when compared with primary CRT in loco-regionally advanced LSCC. The differences of LRC were still well identified on univariate and multivariate analysis when contrasting the two groups based on T stage.

The subgroup analyses showed that cT3 and cT4 cNany primaries treated with surgery had superior LRC compared to primary CRT. In the current series, the OS for surgical and non-surgical groups was similar (63% and 65%) and no statistically significant differences between the two primary treatments of T3 and T4 cNany primaries were noted in this regard. However, the observed LRC difference between surgery and radiotherapy for T3 tumors deviates from the findings reported in the literature. In the Radiation Therapy Oncology Group 91-11 study, which encompassed a patient cohort comprising both T2 and T4 cases, with T3 constituting 80% of the sample, a five-year LRC rate of 67.7% was documented (14).

Among head and neck squamous cell carcinoma, several unique features characterize the laryngeal site. In addition to its fundamental role in breathing and phonation, the larynx works as gatekeeper of the lower airways. These tasks are to be considered, when deciding for functional preservation without affecting tumor control. Different tumor and functional aspects influence the selection of optimal treatment for advanced stage disease in the larynx.

Laryngeal preservation strategies by concomitant CRT for UICC stage III and IV emerged during the last 30 years, strongly supported by results of two landmark randomized trials. The Veterans Affairs Laryngeal Cancer Study (15, 16) and the RTOG91-11 (14, 17) defined the outcome laryngeal preservation based on whether the larynx was removed or not. Both trials, especially the RTOG 91-11 excluded many patients with T4 primaries. The Veterans Affairs Laryngeal Cancer Study resulted in 56% of patients with cT4 primaries (vs. 29% < cT4, p = 0.001) eventually undergoing a salvage laryngectomy (16). The 2-year OS was not different (68% in both arms, p = 0.98) after induction chemotherapy followed by primary RT or primary surgery followed by adjuvant RT. Local recurrences (12% vs. 2%, p = 0.001) were more frequent in the primary RT arm, whereas distant metastases (17% vs. 11%, p = 0.001) and SPM (6% vs. 2%, p = 0.048) were higher after the primary surgery. RTOG 91-11 reported 5-year LRC, PFS and OS as 68%, 31% and 55% in the CRT arm, respectively (14, 17). Our results after CRT are in a similar range.

Several studies gave some hints that non-surgical approaches for larynx-preservation may be associated with decreased OS (18–21). A large series from National Cancer Data Base reported reduced survival in patients with T3 LSCC with the establishment of organ-preservation strategies (18). A retrospective study of locally advanced disease showed that non-surgical larynx-preservation resulted in reduced 5-year OS, particularly for patients with T4 disease (RT: 0%, CRT: 25% and surgery: 55%, p < 0.0001) (19). In a grouped survival data analysis from the Surveillance, Epidemiology, and End Results (SEER) by Megwalu et al. (20), patients with advanced LSCC who underwent surgery had better OS outcomes than those treated with non-surgical approaches (T3: 59% vs. 48%, p < 0.001; T4: 56% vs. 38%, p < 0.001). However, the inherent selection bias in retrospective single institution and national registry studies are well known. The National Cancer Database analysis by Bates et al. (22) demonstrated similar 5-year OS after CRT (53%) and total laryngectomy (49%), p < 0.0001 (p > 0.05 in multivariate analysis). Patients with T4 LSCC showed superior 5-year OS following total laryngectomy (49%) compared to CRT (40%), p < 0.0001. In our series, the OS for surgical and non-surgical groups was similar and no statistically significant differences between the two primary treatments for T3 and T4 cNany primaries were noted (**Table 2**). This remains in line with the study by Timmermans et al. (23) where OS was compared based on treatment modality (total laryngectomy: 52%, RT: 50%, CRT: 43%; p=0.828) and staging (T3: 52% vs. T4: 48%; p=0.528) and showed no difference for either of the two. Concerning the 5-year LRC, our outcome is comparable with the study by Timmermans et al. (23) (total laryngectomy: 87%, CRT: 76%, RT: 65%). Nevertheless, our results in general showed better oncologic outcome than the study by Timmermans et al. (23), which may be caused by the differences of patient and tumor characteristics.

One of the most important factors is the tumor stage corresponding to its extent and expansion pattern. In a retrospective analysis of prospectively collected data, including T3 glottic cancer, tumors <2.5 cc had favorable outcomes, and tumor volume was found to be a significant predictor of survival outcomes (24). In a larger analysis reporting on 9700 patients with advanced (T3-4aN0-3M0), the authors recommend that patients with T4a tumors undergo surgery with aCRT/RT as the preferred initial treatment (25). On the other hand, patients with T3 tumors have the option of either undergoing surgery (followed by aCRT/RT if lymph node involvement is present) or receiving definitive CRT/RT to preserve the larynx. For T4b tumors, being inoperable, the well-accepted management concept is either concomitant CRT or palliative treatment (25). Patients’ quality of life after treating LSCC is highly related to the functional outcome of the organ after treatment. In the absence of well-designed prospective trials reporting the functional outcome and quality of life, several retrospective data were published (26–31). However, laryngeal and hypopharyngeal primaries were usually reported in a combined fashion and heterogeneous outcome parameters were utilized, making cross-comparison between studies difficult. Recently, univariate and multivariate analysis of data from our center reporting on 477 patients who underwent curatively intended treatment for LSCC showed that advanced tumor stage, primary surgery and recurrence are related to a poor functional outcome (32). In this study, 5-year LFS after CRT and primary surgery for stage III-IVB LSCC was 50% and 19%, respectively. As a comparison, Timmermans et al. (23) reported 5-year laryngectomy‐free interval as 72% after RT and 83% after CRT for T3-4 LSCC.

High incidence rates of SPM in head and neck cancer patients remain a major challenge. Cumulatively, about 3% of the successfully treated patients will develop SPM with each passing year (33, 34), which confirms our actuarial incidence rate of 27% within 10 years. Rusthoven et al. (35) reported a significant reduction in SPM incidence in patients who received RT based treatment (hazard ratio: 0.71, 95% confidence interval: 0.65–0.79; p<0.01). Based on the multivariate analysis, these findings were still significant for the laryngeal subsites. In our series and based on only 5-year median follow-up, there was no differences in SPM between the surgical and non-surgical treatment modalities.

The data presented here meet many of the limitations. Besides its retrospective nature, it consists of a heterogeneous population and the treatment offered for surgery group had different procedures such as partial open laryngectomy, total laryngectomy, and transoral laser surgery. Additionally, the primary treatment modality on each patient was decided at the tumorboard and/or based on patients’ preference which inevitably introduces a selection bias. The lack of data on toxicity and the absence of data concerning comorbidities and general condition of the included patients are quite important issues with major impact on the shared decision-making and outcome.

## Conclusion

Our series demonstrated superior LRC with no significant differences in PFS or OS after primary surgery followed by risk-adapted adjuvant (C)RT compared to primary CRT and to the literature in loco-regionally advanced LSCC. The criteria for optimal patient selection to decide which treatment modalities in primary loco-regionally advanced LSCC should be applied still needs to be further defined.

## Data availability statement

The raw data supporting the conclusions of this article will be made available by the authors, without undue reservation.

## Ethics statement

The studies involving human participants were reviewed and approved by Cantonal Ethics Committee of Bern – reference number: 117/14. Written informed consent for participation was not required for this study in accordance with the national legislation and the institutional requirements.

## Author contributions

MS, LA, OE and RG contributed in the conception and design of the study. MS and LA acquired the data. OE performed the statistical analysis. All authors jointly interpreted the data, drafted the work or substantially revised it. All authors approved the submitted version and have agreed both to be personally accountable for the author’s own contributions and do ensure that questions related to the accuracy or integrity of any part of the work, even ones in which each author was not personally involved, are appropriately investigated, resolved, and the resolution is documented in the literature.
